# Associations of urinary polymeric immunoglobulin receptor peptides in the context of cardio-renal syndrome

**DOI:** 10.1038/s41598-020-65154-2

**Published:** 2020-05-19

**Authors:** Tianlin He, Justyna Siwy, Jochen Metzger, William Mullen, Harald Mischak, Joost P. Schanstra, Petra Zürbig, Vera Jankowski

**Affiliations:** 1 Mosaiques Diagnostics GmbH, Hannover, Germany; 2 Institute for Molecular Cardiovascular Research (IMCAR), University Hospital RWTH Aachen, Aachen, Germany; 3 Institute of Cardiovascular and Medical Sciences, University of Glasgow, Glasgow, UK; 4 INSERM U1048, Institute of Cardiovascular and Metabolic Diseases, Toulouse, France; 5 Université Toulouse III Paul-Sabatier, Toulouse, France

**Keywords:** Proteomics, Biomarkers, Cardiology, Nephrology

## Abstract

The polymeric immunoglobulin receptor (pIgR) transports immunoglobulins from the basolateral to the apical surface of epithelial cells. PIgR was recently shown to be associated with kidney dysfunction. The immune defense is initiated at the apical surface of epithelial cells where the N-terminal domain of pIgR, termed secretory component (SC), is proteolytically cleaved and released either unbound (free SC) or bound to immunoglobulins. The aim of our study was to evaluate the association of pIgR peptides with the cardio-renal syndrome in a large cohort and to obtain information on how the SC is released. We investigated urinary peptides of 2964 individuals available in the Human Urine Proteome Database generated using capillary electrophoresis coupled to mass spectrometry. The mean amplitude of 23 different pIgR peptides correlated negatively with the estimated glomerular filtration rate (eGFR, rho = −0.309, p < 0.0001). Furthermore, pIgR peptides were significantly increased in cardiovascular disease (coronary artery disease and heart failure) after adjustment for eGFR. We further predicted potential proteases involved in urinary peptide generation using the Proteasix algorithm. Peptide cleavage site analysis suggested that several, and not one, proteases are involved in the generation of the SC. In this large cohort, we could demonstrate that pIgR is associated with the cardio-renal syndrome and provided a more detailed insight on how pIgR can be potentially cleaved to release the SC.

## Introduction

The polymeric immunoglobulin receptor (pIgR) is a transmembrane protein that is expressed in many mucosal epithelial cell types. PIgR at the basolateral plasma membrane can bind to immunoglobulins of the IgA and IgM isotype. As its main immune defense function, the pIgR transports polymeric immunoglobulins (pIgs) across epithelia to mucosal secretions at the apical compartment where pIgs are cleaved off from pIgR, leaving a fragment of pIgR called secretory component (SC) covalently attached to the secreted pIgs. Therefore, SC is directly involved in the protective function of secretory IgA. In addition, free SC exhibits scavenger properties with respect to enteric pathogens^1^.

In general, the glycosylated pIgR is a single pass type I receptor and consists of an extracellular and a transmembrane region and a cytoplasmic tail^2^. The extracellular immunoglobulin-binding region is further divided into five domains of around 100 amino acids which are highly conserved^3^. A sixth more distantly related domain follows these five and includes the transmembrane region of the molecule^2^. This sixth domain is involved in the enzymatic cleavage and release of the pIgR into the intestinal lumen as SC. However, the mechanism by which the pIgR is cleaved into the SC remains elusive^4^. The prediction of a consensus cleavage site is hampered, because the sequence and the length of the linker peptide is poorly conserved across species and the C-terminus of the SC has not been identified^5^. Therefore, the identity of the protease that cleaves pIgR to SC remains unknown to date.

Cytoplasmic and membranous expression of the pIgR was observed mostly in the gall- and urinary bladder, kidney, and gastrointestinal mucosa^6^. However, in contrast to studies of organs like the intestine, the regulation of pIgR in human kidneys was, until previously, incompletely investigated. In a recent study, Krawczyk *et al*. investigated the localization and regulation of pIgR in human kidneys^7^. They were able to show that human pIgR is expressed in proximal tubules and glomeruli. Furthermore, they demonstrated that during chronic kidney disease (CKD) the expression of pIgR in the tubules becomes prevalent, associated with increased levels of urinary secretory immunoglobulins. Their results indicated that elevated pIgR expression is a substantial part of the tubular response to injury, providing an explanation for the increased levels of secreted pIgs found in urine from injured kidneys.

Due to the fact that heart and kidneys are involved in maintaining hemodynamic stability and organ perfusion through an intricate network, the pIgR might also be associated to coronary artery diseases (CAD). However, the only link between pIgR and atherosclerosis is the elevated level of this protein in extracellular vesicles from individuals with acute coronary syndrome, although inclusion of this parameter did not improve disease detection over conventional risk factors or troponin I^8^. Elevated levels of total serum IgA have been reported in patients with severe atherosclerosis or with previous myocardial infarction or other major ischemic events^9,10^.

Therefore, the aim of this study was to investigate whether altered pIgR expression is associated with cardio-renal syndrome using urinary proteome analysis. In addition, the analysis of endogenous urinary peptides could provide further information about the potential cleavage of pIgR to release the SC.

## Results

### Release of pIgR peptides in urine

**Figure 1:**
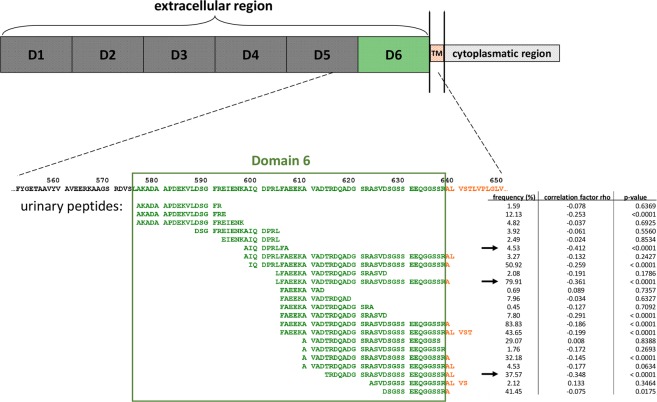
Identified endogenous urinary pIgR peptides and their position in the pIgR protein sequence. D1-D6: Domain 1–6; TM: transmembrane region. The table shows the frequency of each individual peptide in the analysed samples, the correlation factor rho with estimated glomerular filtration rate (eGFR) and the associated p-value. Arrows indicate the individual peptides with the highest negative correlations with eGFR.

We investigated urinary pIgR peptides which were identified previously and stored in the Human Urinary Proteome database^11^. In total, 23 different pIgR-derived naturally occurring peptides were found in urine by proteome analysis. These peptides showed a high degree of sequence overlap, covering mainly domain 6 of the extracellular region (Fig. 1, green) and the first amino acids (AA) from the transmembrane region (Fig. 1, orange) in the pIgR protein (from AA 576 to 648). Most of the peptides showed an inconspicuous cleavage site at their C-terminus and some also at their N-terminus. However, there were 6 pIgR-peptides with their N-terminus at AA position 605 and 2 peptides with a C-terminus at AA position 604, which are indicative for a specific proteolytic cleavage site. However, there seem to be further cleavage sites before AA positions 610 and 576.

In order to predict the proteases possibly involved in the generation of the urinary pIgR-peptides, *in-silico* protease prediction was performed with the open-source tool Proteasix^12^. By using this bioinformatics approach on the naturally occurring peptides as cleaved end products it is possible to track back to the enzymes potentially responsible for the generation of these peptides. The majority of human proteases have several protein targets and therefore one peptide sequence might be cleaved by different proteases. The analysis yielded a list of 52 endopeptidases putatively responsible for the generation of the identified pIgR-peptides in urine (Supplementary Table 1). From the 52 predicted proteases, 12 proteases were most frequently selected (predicted protease in ≥ 50% of total cleavage assignments) in the Proteasix analysis including calpains CAPN1 and CAPN2, cathepsins CTSB, CTSD and CTSG, matrix metalloproteinases MMP14, MMP25, MMP3, MMP7 and MMP9, as well as pepsin A-3 (PEPA3) and meprin A subunit alpha (MEP1A). Furthermore, when focussing on the three potential cleavage sites of the pIgR-protein for the release of the SC (mentioned above) CAPN1, CAPN2, CTSD, CTSG, MMP3, MMP7, MMP9, PEPA3, and MEP1A were most frequently predicted.

### Association of urinary pIgR peptides with estimated glomerular filtration rate (eGFR)

**Figure 2:**
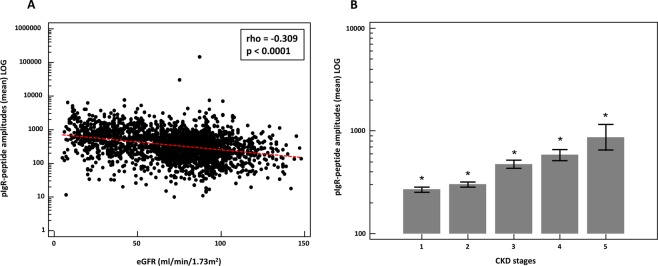
(**A**) Scatter diagram of the correlation between mean amplitudes of the pIgR peptides (LOG) and estimated glomerular filtration rate (eGFR). (**B**) Mean pIgR peptide amplitudes (±95% CI) in urine distributed to the different CKD stages. *Indicates p < 0.05 in comparison to all different CKD stages.

To investigate the association of these pIgR-peptides with kidney function, we used 2449 relevant (investigated in the context of CKD) proteome datasets with patient information on their eGFR. Based on the assumption that these peptides depict the whole spectrum of pIgR protein abundance, we calculated the mean amplitude of all peptides in each CKD aetiology and healthy control datasets. When investigating association of mean pIgR abundance with eGFR of the total cohort, a significant negative correlation was detected (rho = −0.309, p < 0.0001) as shown in Fig. 2A. The correlation analysis without the controls (n = 1243; rho = −0.376) is even significantly better (p = 0.029) compared to the correlation with the controls. Assessment of the correlation of the individual peptides in all individuals showed that 10 out of the 23 peptides contributed to this correlation (table in Fig. 1). However, these individual significant correlations were very variable ranging from rho = −0.075 to -0.412. Some of the peptides displayed a stronger negative correlation than the correlation of the combined peptides including peptide AA 598–606 (rho = −0.412), AA 604–639 (rho = −0.361), and AA 614–640 (rho = −0.348) (black arrows in Fig. 1). We also investigated the correlation of the pIgR peptides with urinary albumin concentrations which was positive (rho = 0.210; p < 0.0001). Finally, we looked at the pIgR amplitudes divided into the different CKD stages. As depicted in Fig. 2B, the combined pIgR peptide amplitudes were significantly increased with higher CKD stages.

**Figure 3:**
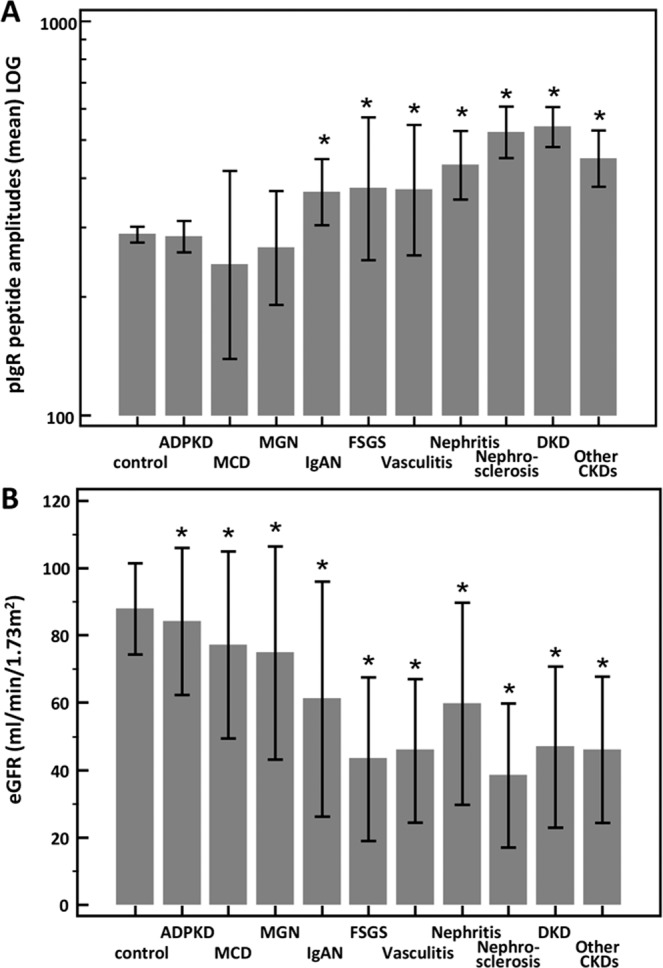
(**A**) Mean pIgR peptide amplitudes (±95% CI) and (**B**) estimated glomerular filtration rate (eGFR) (±SD) in urine of patients with different kidney disease conditions and healthy controls. *Indicates p < 0.05 in comparison to healthy controls. ADPKD: autosomal dominant polycystic kidney disease, MCD: minimal change disease, MGN: membranous glomerulonephritis, IgAN: IgA nephropathy, FSGS: focal segmental glomerulosclerosis, DKD: diabetic kidney disease.

After the investigation of the association of the pIgR-peptides with the eGFR in the full cohort, we also investigated their mean amplitude in different kidney disease etiologies. We investigated the following CKDs: autosomal dominant polycystic kidney disease (ADPKD), minimal change disease (MCD), membranous glomerulonephritis (MGN), IgA nephropathy (IgAN), focal segmental glomerulosclerosis (FSGS), ANCA-associated vasculitis, nephritis, nephrosclerosis, diabetic kidney disease (DKD), and other CKDs (e.g. solitary kidneys, other glomerular diseases, urologic/reflux nephropathy, cystic kidneys). As demonstrated in Fig. 3A, the lowest mean amplitude of pIgR is observed in urine of patients with MCD and the highest levels are in the urine of patients suffering from DKD. However, the differences between the mean amplitude of pIgR-peptides in healthy controls and CKDs differs significantly only with the disease groups IgAN, FSGS, vasculitis, nephritis, nephrosclerosis, DKD, and all other CKDs. In addition, as observed above, although with some exceptions, inverse association between the pIgR-peptide mean amplitude and eGFR is evident (Fig. 3B).

In addition, we performed further analyses with Proteasix by using only the pIgR peptides which were significantly associated with eGFR in each CKD aetiology and predicted the proteases preferentially involved in cleavage of those peptides (Supplementary Table 2). The following potential proteases for the different CKD aetiologies were identified: ADPKD (MMP7), MCD (CTSB, CTSD, CTSE, MEP1A, MMP7, PGA3), MGN (MMP3, MMP7, MMP9, MMP13, MMP14), IgAN (MMP7), FSGS (MMP3, MMP7), vasculitis (PGA3), nephritis (MMP7, MMP9), nephrosclerosis (MMP7), and DKD (MMP9).

### Regulation of urinary pIgR peptides in cardiovascular diseases

**Figure 4:**
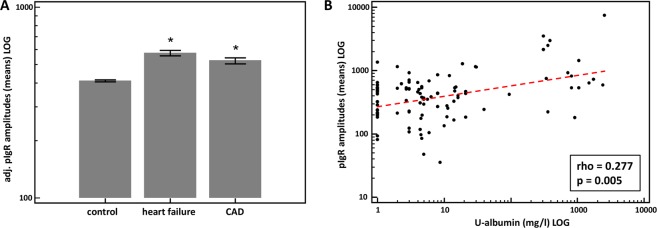
(**A**) Adjusted (for eGFR) mean pIgR peptide amplitudes (±95% CI) in urine of patients with different cardiovascular disease conditions (CAD and heart failure) and healthy controls. (**B**) Scatter diagram of the correlation between mean amplitudes of the pIgR peptides (LOG) and urinary albumin excretion (LOG) in patients with cardiovascular disease conditions and with an eGFR > 60 ml/min/1.73m^2^. *Indicates p < 0.05 in comparison to healthy controls. CAD: coronary artery disease.

To investigate the association of pIgR peptides with cardiovascular diseases, we extracted additional urinary proteome data from the Human Urinary Database with respect to heart failure and CAD with patient information on the eGFR. We compared the urinary levels of pIgR peptides in these CAD with those of the healthy controls. To eliminate the influence of eGFR on the pIgR levels, we adjusted the proteome data for this parameter with the use of multiple regression analysis. As shown in Fig. 4A, the levels of pIgR peptides in patients with heart failure and CAD when compared with healthy controls are significantly increased. Furthermore, we investigated the urinary albumin excretion, which can also be a risk factor for cardiovascular disease. We used a data subset of CVD patients with an eGFR > 60 ml/min/1.73m^2^ (n = 101). There was a significant positive correlation of albumin with pIgR peptides levels (rho = 0.277; p = 0.005) in the heart failure and CAD patients without CKD (see Fig. 4B).

## Discussion

Based on a large dataset representing approximately 3000 patients, our data support the results by Krawczyk *et al*. that urinary SC levels of the pIgR protein are positively correlated to the severity of kidney injury in patients with kidney disease^7^. We could further confirm the findings reported by Krawczyk *et al*. that the pIgR amplitude is lower (but not significantly) in the urine of MCD patients than it is in healthy controls. Finally, some pIgR peptides are also included in the peptide-based classifier CKD273 which was proven in several studies to have a high accuracy in the early diagnosis and the prediction of CKD progression^13,14^. Therefore, the current study confirms the association of pIgR peptides with renal (dys)function. Furthermore, we were able to demonstrate that there is also an association of pIgR with CAD and heart failure. Due to the fact that pIgR/SC is not expressed in heart cells^15^, our findings point towards inflammatory pathways with respect to cardiovascular disease risk. A first hint in this direction was shown in a study where pIgR is associated with coronary atherosclerosis in coronary vessels of individuals chronically exposed to increased ambient concentrations of traffic air pollution^16^. In general, we showed with this study that the large collection of clinical and proteomics data available the Human Urinary Proteome Database can be of great use for the evaluation of specific urinary peptide levels under different disease conditions.

In addition, the identification of naturally occurring urinary pIgR peptides may also provide further insights into the structure of the SC of the pIgR protein. First, the vast majority of the identified endogenous peptides are derived from domain 6 of the extracellular region in the pIgR protein (see Fig. 1), as described in the study of Sunagawa *et al*.^17^. Interestingly, the lowest AA number of the identified urinary peptides starts at position 576 of the pIgR protein and not at position 575. Furthermore, the identified urinary pIgR peptides with the highest AA number end at position 643 which means that the SC also includes parts of the suggested transmembrane region^3^. This confirms the observation that based on cloning and sequencing of DNA complementary to pIgR mRNA isolated from rabbit liver, the sixth domain also included a portion of the membrane-spanning domain^2^.

Several groups tried to determine the exact cleavage site for the secretion of SC^5,18,19^. In a study using a recombinant vaccinia virus-mediated transient expression system, the importance of domain 6 of the pIgR for cleavage of the receptor was demonstrated^19^. A highly conserved 9-amino acid sequence is present in this region in various species^17^. This 9-amino acid sequence FAEEKAVAD ranging from position 605 to position 613 in pIgR is predicted to encode an α-helical region that is critical for pIgR cleavage^17^. In our study, we found that 10 of the 24 different urinary peptides include these 9 AAs. Furthermore, based on the peptide sequences it seems that there are at least 3 cleavage sites specific for calpains, cathepsins, matrix metalloproteinases, pepsin A-3, and meprin A subunit alpha. These results confirm the hypothesis from Kaetzel *et al*. that more than just one potential protease enables the release of the SC^4^. Regulation via differential protease activity seems necessary to maintain tissue homeostasis and directly linked to the generation of pIgR peptides associated with CKD^20,21^. Bioinformatics peptide centric tools have been developed in order to determine which proteases are potentially responsible for the changes in the urinary peptidome during CKD^12,22^. In the present study, we were able to specify several potential proteases for the generation of the identified urinary pIgR-peptides which belong to the family of metalloproteinases, cathepsins, calpains, mephrins, and pepsins. Out of these, four were already described to be deregulated in CKD patients: MMP3, MMP7, MMP9, and CTSD^20^. The CKD aetiology-based protease prediction suggested some relatively specific proteases for the different CDK aetiologies which remains to be confirmed. The dysregulation of matrix metalloproteinases has been linked to renal fibrosis progression^23^, as well as to CAD^24^. CTSD, also verified in a previous study, is known to mediate inflammation^25^ and was reported to be in increased levels in human kidney tissue of patients with CKD, especially in the areas of tubular damage^26^. Thus, the findings of protease dysregulation support an imbalance of inflammatory and fibrotic processes in CKD^22^. However, the predicted protease activity has to be confirmed by lab-based generated evidence.

In conclusion, based on data focussing on urinary peptides in a large cohort of almost 3000 individuals, this study supports recent findings that pIgR is correlated with changes in kidney function and with an association to coronary artery disease. Furthermore, this study provides some first hints into the mechanisms how pIgR can potentially be cleaved to release the SC.

## Materials and Methods

For this study urinary proteome data stored in the Human Urine Proteome Database^11^ obtained by capillary electrophoresis coupled to mass spectrometry was used. This database available at Mosaiques Diagnostics (Hannover, Germany) includes anonymized clinical information of participants enrolled in several studies as well as urinary proteomic data. The study was approved by the ethics committee from Hannover Medical School, Germany (ID 3116–2016), fulfilling all the requirements on the protection of the individuals participating in medical research and in accordance with the principles of the Declaration of Helsinki^27^. All data sets received were anonymized. Urine samples were collected from 20 different centres: Steno Diabetes Center (Gentofte, Denmark; n = 67); Austin Health and Northern Health, University of Melbourne, (Melbourne, Australia; n = 20); Hannover Medical School (Hannover, Germany; n = 380); Charité (Berlin, Germany; n = 105); RWTH Hospital Aachen (Aachen, Germany; n = 54); LMU Munich (Munich, Germany; n = 70); Technical University of Munich (Munich, Germany; n = 8); Human Nutrition and Metabolism Research and Training Center, Karl Franzens University of Graz (Graz, Austria; n = 37); Barbara Davis Center for Childhood Diabetes, University of Colorado Denver (Denver, USA; n = 31); University of Virginia (Charlottesville, Virginia, USA; n = 10); University of Alabama (Birmingham, Alabama, USA; n = 17); Charles University (Prague, Czech Republic; n = 28); University of Skopje (Skopje, Macedonia; n = 127); University Medical Center (Groningen, The Netherlands; n = 46); INSERM (Toulouse, France; n = 65); RD-Néphrologie, Néphrologie Dialyse St. Guilhem (Montpellier, France; n = 42); University of Leuven (Leuven, Belgium; n = 488); Ghent University (Ghent, Belgium; n = 393); University Hospital Zürich (Zürich, Switzerland; n = 268); and BHF Glasgow Cardiovascular Research Centre (Glasgow, UK; n = 451). Sample collection was performed in accordance to local ethics requirements and all individuals gave written informed consent. The total number of datasets used for this study was n = 2707.

The estimated glomerular filtration rate (eGFR) was calculated from serum creatinine using the Chronic Kidney Disease Epidemiology Collaboration (CKD-EPI) equation^28^. Table 1 summarizes patient numbers, eGFR values, and pIgR peptide amplitudes in the different disease conditions.

**Table 1:** Patients data in the different disease conditions.

Diagnosis	cohort	sex	age (years)		eGFR (ml/min/1.73 m^2^)		u-albumin (mg/L) [LOG]		pIgR-peptide amplitude [LOG]	
	(n)	(male %)	mean	SD	mean	SD	geom. mean	95% CI	geom. mean	95% CI
Healthy control	1206	53	48	17	88	14	1.29	1.22–1.37	287.20	274.31–300.70
ADPKD	299	46	33	9	84	22	28.07	19.16–41.12	283.84	259.07–310.97
MCD	26	77	46	19	77	28	572.93	90.15–3640.95	240.93	139.33–416.64
MGN	50	72	53	14	75	32	666.95	346.39–1284.19	266.03	190.53–371.45
IgAN	114	67	45	15	61	35	283.85	185.96–433.28	368.64	304.07–446.93
FSGS	47	60	47	19	43	24	376.15	166.41–850.26	375.95	247.80–570.39
Vasculitis	43	51	65	9	46	21	203.83	135.34–306.97	373.15	254.51–547.09
Nephritis	115	36	49	18	60	30	122.77	75.36–200.00	430.80	352.44–526.57
Nephrosclerosis	133	66	67	14	38	21	43.39	28.60–65.82	522.50	450.12–606.52
DKD	304	65	64	12	47	24	187.59	153.74–228.91	538.50	479.60–604.64
other CKDs	112	58	60	17	45	18	29.66	21.90–40.17	448.04	378.88–529.82
CAD	263	67	64	11	56	27	18.87	13.80–25.80	434.26	384.79–490.09
Heart failure	252	61	67	12	65	25	26.76	13.71–52.24	378.06	340.23–420.10

ADPKD: autosomal dominant polycystic kidney disease, MCD: minimal change disease, MGN: membranous glomerulonephritis, IgAN: IgA nephropathy, FSGS: focal segmental glomerulosclerosis, DKD: diabetic kidney disease, CKD: chronic kidney disease, CAD: coronary artery disease.

## Protease Prediction

The open-source tool for protease prediction – Proteasix (www.proteasix.org) was used for the analysis to link naturally occurring peptides in urine to the proteases potentially involved in their generation^12^. Proteasix uses information about naturally occurring peptides i.e. corresponding protein UniProt ID and start/stop amino acid position to predict potential cleaving proteases. Proteasix retrieves information about cleavage sites from protease databases (MEROPS, BRENDA) considering also cleavage site restrictions (from ENZYME database). A list of predicted proteases is generated as a result of the analysis.

### Statistical methods

For statistical analysis MedCalc software version 12.7.5.0 (SAS Institute, INC., Cary; NC, USA) was used. Because the amplitudes of the peptides across the samples are not normally distributed, we used the nonparametric Spearman’s rank correlation coefficient (rho) for estimating the correlation of pIgR peptides with eGFR or urinary albumin. Means were compared using one-way ANOVA followed by Tukey-Kramer test for multiple comparisons. The pIgR levels were adjusted for eGFR with multiple regression analysis. A p-value of 0.05 was selected as the significance level.

## Supplementary information

Supplementary information.

Supplementary table 1.

Supplementary table 2.

## Data Availability

The datasets generated during and/or analysed during the current study are available from the corresponding author on reasonable request.
