# Supplementary material for: Associations of urinary polymeric immunoglobulin receptor peptides in the context of cardio-renal syndrome
Source: Sci Rep. 2020 May 19;10:8291. doi: 10.1038/s41598-020-65154-2 (PMC7237418; doi:10.1038/s41598-020-65154-2)
Supplement: Supplementary file 1 — Supplementary information. [file 41598_2020_65154_MOESM1_ESM.doc]

**Associations of urinary polymeric immunoglobulin receptor peptides in the context of cardio-renal syndrome**

Tianlin He^1,2^, Justyna Siwy^1^, Jochen Metzger^1^, William Mullen^3^, Harald Mischak^1,3^, Joost P. Schanstra^4,5,*^, Petra Zürbig^1,*^, Vera Jankowski^2^

^1^ Mosaiques Diagnostics GmbH, Hannover, Germany

^2^ Institute for Molecular Cardiovascular Research (IMCAR), University Hospital RWTH Aachen, Germany

^3^ Institute of Cardiovascular and Medical Sciences University of Glasgow, Glasgow, UK

^4^ INSERM U1048, Institute of Cardiovascular and Metabolic Diseases, Toulouse, France

^5^ Université Toulouse III Paul-Sabatier, Toulouse, France

**Supplementary data**

**Supplementary methods**

This is a detailed description of the previously performed urinary proteome analysis as a basis for the Urinary Proteome Database.

**Preparation and processing of the urine samples**

Urine (0.7 mL) was thawed immediately before analysis and diluted with 0.7 mL of 2 M urea, 10 mM NH_4_OH containing 0.02% SDS [1]. To remove higher molecular mass proteins, such as albumin and immunoglobulin G, the sample was ultra-filtered using Centrisart ultracentrifugation devices (20 kDa MWCO; Sartorius, Göttingen, Germany) at 3,000 g relative centrifugal force until 1.1 mL of filtrate was obtained. This filtrate was then applied onto a PD-10 desalting column (GE Healthcare, Uppsala, Sweden) equilibrated in 0.01% NH_4_OH in HPLC-grade H_2_O (Roth, Germany) to decrease matrix effects by removing urea, electrolytes, and salts, and to enrich polypeptides. Finally, all samples were lyophilized, stored at 4 ºC, and suspended in HPLC-grade H_2_O shortly before CE-MS analyses [2].

CE-MS analyses were performed using a P/ACE MDQ capillary electrophoresis system (Beckman Coulter, Fullerton, USA) on-line coupled to a micrOTOF MS (Bruker Daltonic, Bremen, Germany) [2]. The ESI sprayer (Agilent Technologies, Palo Alto, CA, USA) was grounded, and the ion spray interface potential was set between ‑4 and ‑4.5 kV. Data acquisition and mass spectrometry acquisition methods were automatically controlled by the capillary electrophoresis via contact-close-relays. Spectra were accumulated every 3 s, over a range of charge states (m/z) 350 to 3000.

Mass spectra were processed using MosaiquesVisu software, including peak picking, deconvolution and deisotoping [3]. Mass deviation will be <25 ppm for monoisotopic resolution and <100 ppm for unresolved peaks (z>6). To ensure high data consistency, one sample data set has to consist of a minimum of 800 peptides with a minimal MS-resolution of 8000 and a minimal migration time interval of 10 min. The limit of detection will be ~1 fmol. Previous publications described the accuracy, precision, selectivity, sensitivity, reproducibility, and stability of the CE-MS measurements in detail [4, 5].

Migration time and peak intensity were normalized using internal peptide standards [6]. Reference signals of 1770 urinary peptides will be used for CE-time calibration by local regression. For normalization of analytical and urine dilution variances, MS signal intensities will be normalized relative to 29 internal standard peptides generally present in at least 90% of all urine samples with small relative standard deviation. For calibration, linear regression will be performed. The resulting peak list characterizes each peptide by its molecular mass, normalized capillary electrophoresis migration time, and normalized signal intensity. Normalized signal intensity is used as a measure for relative abundance. The entire analytical process is also graphically depicted in **figure A**. All detected peptides were deposited, matched, and annotated in the Microsoft SQL database (Human Urinary Proteome Database) [7,8], allowing further analysis and comparison of multiple patient groups.


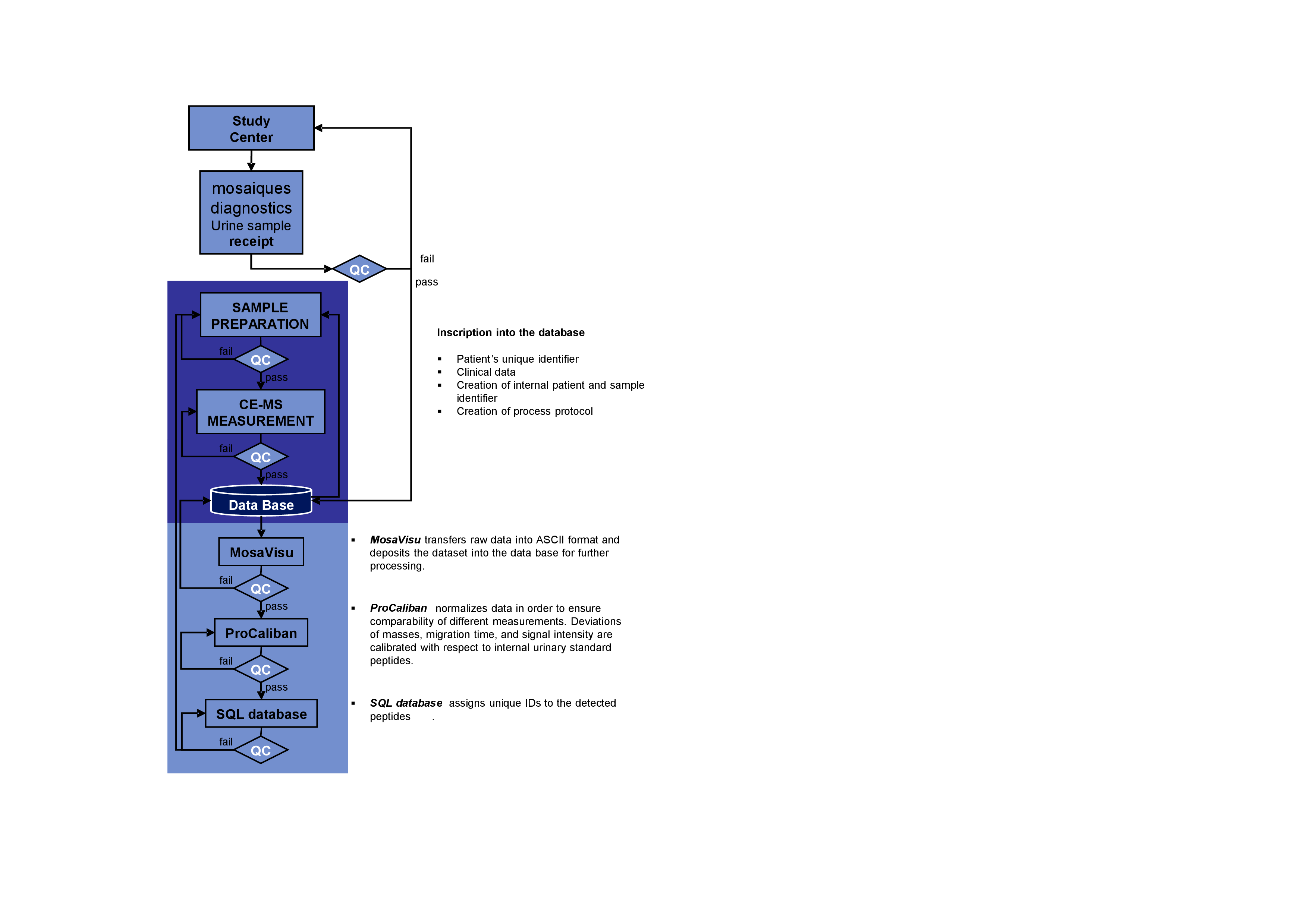


**Figure A.** Graphic depiction of CE-MS analysis and subsequent data evaluation.

**Peptide sequence analysis**

Peptide sequencing was performed using Dionex Ultimate 3000 RSLS nano flow system (Dionex, Camberly,UK) or Beckman CE, coupled to an Orbitrap Velos MS instrument (Thermo Scientific,Waltham, Massachusetts, US) [9]. The obtained spectra were analysed with Proteome Discoverer 1.4 (Thermo Scientific) (with precursor mass tolerance of 5 pmm and fragment mass tolerance of 0.05 Da) and searched against UniProt human non-redundant database. Oxidation of methionine and proline were considered as variable modifications. Criteria for sequence acceptance were high confidence score (Xcorr ≥ 1.9) and lack of unmodified cysteine. To prevent false identification of sequences, a strong correlation between peptide charge at the working pH of 2 and capillary electrophoresis migration time was used [10].

**Supplementary table legends**

**Supplementary table 1.** Proteases predicted by Proteasix software based on the input list of 23 differentially expressed pIgR peptides.

**Supplementary table 2.** Proteases predicted by Proteasix software based on the input list of significantly with eGFR correlated pIgR peptides in each CKD aetiology.

**References**

1. Theodorescu, D. *et al*. Pilot study of capillary electrophoresis coupled to mass spectrometry as a tool to define potential prostate cancer biomarkers in urine. Electrophoresis 2005; 26: 2797-2808.

2. Theodorescu, D. *et al*. Discovery and validation of new protein biomarkers for urothelial cancer: a prospective analysis. Lancet Oncol 2006; 7: 230-240.

3. Neuhoff, N. V. *et al*. Mass spectrometry for the detection of differentially expressed proteins: a comparison of surface-enhanced laser desorption/ionization and capillary electrophoresis/mass spectrometry. Rapid Commun Mass Spectrom 2004; 18: 149-156.

4. Good, D. M. *et al.* Naturally occurring human urinary peptides for use in diagnosis of chronic kidney disease. MCP 2010, 9, 2424-2437.

5. Mischak, H. *et al.* Technical aspects and inter-laboratory variability in native peptide profiling: the CE-MS experience. Clin Biochem 2013, 46(6), 432-443.

6. Jantos-Siwy, J. *et al*. Quantitative urinary proteome analysis for biomarker evaluation in chronic kidney disease. J Proteome Res 2009; 8: 268-281.

7. Coon, J. J. *et al*. CE-MS analysis of the human urinary proteome for biomarker discovery and disease diagnostics. Proteomics Clin Appl 2008; 2: 964.

8. Siwy, J. *et al.* Human urinary peptide database for multiple disease biomarker discovery. Proteomics Clin Appl 2011, 5, 367-374.

9. Klein, J., *et al.* Comparison of CE-MS/MS and LC-MS/MS sequencing demonstrates significant complementarity in natural peptide identification in human urine. Electrophoresis 2014, 35, 1060-1064.

10. Zürbig, P. *et al.* Biomarker discovery by CE-MS enables sequence analysis via MS/MS with platform-independent separation. Electrophoresis 2006, 27, 2111-2125.
